# All-optical generation and ultrafast tuning of non-linear spin Hall current

**DOI:** 10.1038/s41598-018-35378-4

**Published:** 2018-11-20

**Authors:** Jonas Wätzel, Jamal Berakdar

**Affiliations:** Institute for Physics, Martin-Luther-University Halle-Wittenberg, 06099 Halle, Germany

## Abstract

Spin Hall effect, one of the cornerstones in spintronics refers to the emergence of an imbalance in the spin density transverse to a charge flow in a sample under voltage bias. This study points to a novel way for an ultrafast generation and tuning of a unidirectional nonlinear spin Hall current by means of subpicosecond laser pulses of optical vortices. When interacting with matter, the optical orbital angular momentum (OAM) carried by the vortex and quantified by its topological charge is transferred to the charge carriers. The residual spin-orbital coupling in the sample together with confinement effects allow exploiting the absorbed optical OAM for spatio-temporally controlling the spin channels. Both the non-linear spin Hall current and the dynamical spin Hall angle increase for a higher optical topological charge. The reason is the transfer of a higher amount of OAM and the enhancement of the effective spin-orbit interaction strength. No bias voltage is needed. We demonstrate that the spin Hall current can be all-optically generated in an open circuit geometry for ring-structured samples. These results follow from a full-fledged propagation of the spin-dependent quantum dynamics on a time-space grid coupled to the phononic environment. The findings point to a versatile and controllable tool for the ultrafast generation of spin accumulations with a variety of applications such as a source for ultrafast spin transfer torque and charge and spin current pulse emitter.

## Introduction

A key issue in spintronics, a field^1–3^ that utilizes spin dynamics for information processing and storage, has been to tweak the spins by electrical means by applying for instance electric gates or voltage pulses. The promise is a swift and energy-saving operation as compared to using magnetic fields as well as opening the door for a wider class of materials. A key to electrical manipulation of spin is the spin-orbit interaction (SOI) which can be substantially enhanced for low dimensional structures dictating, for example, new phases at interfaces^4–7^.

One of the most intensively researched SOI-induced phenomenona is the spin Hall effect (SHE)^8–14^. SHE has important applications, for instance, it renders possible the generation and detection of spin currents^15–18^, and it contributes with a spin-torque that can be used to steer magnetization dynamics^19–21^. The issue of how fast, a possibly non-linear SHE can be triggered raises naturally the question of whether a unidirectional spin Hall current can be generated optically on an ultrafast (THz) timescale. Generically, the electric field component of the optical pulse couples to the carriers orbital motion and the spin is affected via SOI. Photo-induced unidirectional currents, needed for SHE, requires, however, an appropriate break of the underlying symmetry^14^ which is reflected in a certain topology of the electronic structure posing so constraint on the class of materials that can be employed.

Here we will follow a different route to generate and enhance photocurrent by exploiting the topology of the light fields, meaning the wave front of the optical field (instead of the sample) is assumed to be prepared to have the appropriate symmetry. Below we employ optical vortices that carry (and impart when interacting with matter) a tunable amount of orbital angular momentum (OAM) related to the topological charge of the vortex^22–29^. Various methods exist for the generation of optical vortices. To name a few, spatial light modulators (SLM)^30,31^ and (optimized) spiral phase plates^29,32^ were utilized as well as metasurfaces^33^ allowing winding numbers *m*_OAM_ > 10 for a wide range of parameters. Various techniques have advantages and limitations: The usage of SLM allows a dynamical control of the generated OAM light, while the efficiency is relatively low and the beam quality is restricted by the pixel size of the used nematic liquid crystal cells. In contrast, spiral phase plates and metasurfaces generate accurate optical vortices but are static approaches. Recent advances in nano-fabrication and engineering of optical materials, nano-scaled integrated OAM laser devices offer versatile tools for shaping the beams to carry well defined and adjustable OAM^34^.

**Figure 1:**
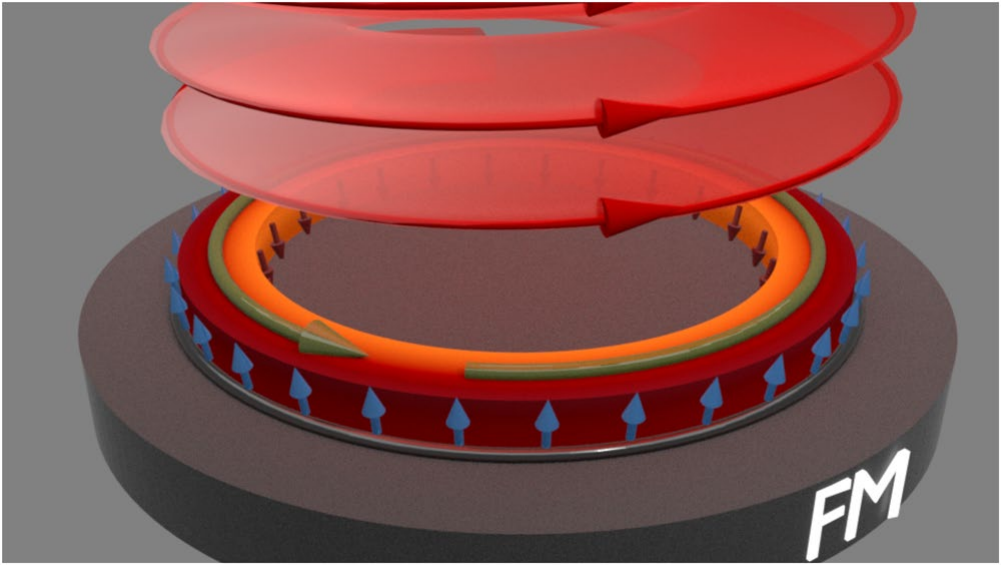
Schematics of the optically induced unidirectional spin Hall current. A focused THz optical vortex beam (red circles) initiates a unidirectional circulating current ***j*** (green arrow) upon transfer of optical OAM to charge carriers. Due to SOI, the angular orbital motion is accompanied by an drift spin-polarized (Hall) current leading to accumulation of spin-polarized charge density (indicated by arrows) at the ring boundaries which can act with a torque on an interfaced magnet.

In the context of this work, experiments on solid (semi-conducting) samples evidence the light-matter OAM transfer^35–37^. Below, we demonstrate how tuning the optical OAM enhances the photocurrent and effectively increases the residual SOI (we focus on intrinsic contributions to SHE). For all-optical devices, we generate the photo-current in an open circuit geometry by employing a ring structure, as depicted in Fig. 1. The optical vortex whirls the charge density around the ring in a steady-state with a well-defined direction set by the sign of the vortex topological charge. Assisted by SOI, the photocurrent results in a substantial, robust SHE with an associated spin torque that can be, for instance, used to influence the magnetic dynamics in an adjusted magnetic layer, as schematically illustrated in Fig. 1. The effect can be triggered and controlled on a picosecond time scale, and its magnitude and direction are tunable with the OAM of the optical vortex.

## Results

### Setup and theoretical modelling

For the emergence of photo-induced SHE the SOI is crucial. For considered systems, the Dresselhaus SOI arises from the crystal lattice inversion asymmetry^38^ while the Rashba SOI is induced by the structure inversion-asymmetry^39^ and depends strongly on the electrical environment, for instance, its effective strength can be tuned by a gate voltage^40^. Previous studies considered the time-dependent charge polarization in low-dimensional nano-structures^41–54^, to our knowledge, however, spin current generation with optical vortices has not been studied so far. To be specific, we consider in Fig. 1 the intra-conduction band carrier dynamics in an appropriately doped InGaAs-InAs-InAlAs-based ring^55–57^ that define the *x* − *y* plane. The rings are irradiated by an optical vortex pulse propagating in *z*-direction and tightly focused on the ring. Rashba SOI is substantial^58^. The vertical confinement potential *U*(*z*) in the growth direction is such that only the lowest subband is involved, meaning the laser frequency is tuned to the regime *ω*_*x*_ < 3*ħ*^2^/(2m^*^Δ*h*) where Δ*h* is the ring height in the *z* direction, and m^*^ = 0.023*m*_*e*_ is the effective mass^59^. Thus, we focus on the spin-dependent charge dynamics in the *x* − *y* plane is the presence of the laser. Adopting polar coordinates {*r*, *φ*}, in the *x* − *y* plane the carriers move the azimuthal direction ${\hat{\varepsilon }}_{\phi }$ while being restricted radially by the confinement potential *V*(*r*). The effective single-particle Hamiltonian reads

1 $$\hat{H}(t)={\hat{H}}_{0}+{\hat{H}}_{{\rm{i}}{\rm{n}}{\rm{t}}}(t)+{\hat{H}}_{{\rm{p}}{\rm{h}}}+{\hat{H}}_{{\rm{b}}{\rm{a}}{\rm{t}}{\rm{h}}}$$

where the unperturbed carriers Hamiltonian ${\hat{H}}_{0}$, the coupling to the laser fields ${\hat{H}}_{{\rm{int}}}(t)$, as well as the phononic environment ${\hat{H}}_{{\rm{ph}}}$ and ${\hat{H}}_{{\rm{b}}{\rm{a}}{\rm{t}}{\rm{h}}}$ are given respectively by

2 $$\begin{array}{rcl}{\hat{H}}_{0} & = & \frac{{\hat{p}}^{2}}{2{m}^{\ast }}+V(r)+\frac{\alpha }{\hslash }{[\hat{\sigma }\times \hat{p}]}_{z}\\ {\hat{H}}_{{\rm{int}}}(t) & = & \frac{e}{2{m}^{\ast }}[\hat{{\boldsymbol{p}}}\cdot {\boldsymbol{A}}({\boldsymbol{r}},t)+{\boldsymbol{A}}({\boldsymbol{r}},t)\cdot \hat{{\boldsymbol{p}}}+e{\boldsymbol{A}}{({\boldsymbol{r}},t)}^{2}]\\  &  & +\,\frac{e\alpha }{\hslash }{[\hat{\sigma }\times {\boldsymbol{A}}({\boldsymbol{r}},t)]}_{z}+e{\rm{\Phi }}({\boldsymbol{r}},t)\\ {\hat{H}}_{{\rm{bath}}} & = & \sum _{{\boldsymbol{q}},\lambda }\,\hslash {\omega }_{{\boldsymbol{q}},\lambda }\,{b}_{{\boldsymbol{q}}\lambda }^{\dagger }{b}_{{\boldsymbol{q}}\lambda }\\ {\hat{H}}_{{\rm{ph}}} & = & \sum _{{\boldsymbol{q}},\lambda }{M}_{\lambda }({\boldsymbol{q}})({b}_{{\boldsymbol{q}}\lambda }^{\dagger }\,\exp \,(\,-\,i{\boldsymbol{q}}\cdot {\boldsymbol{r}})+{b}_{{\boldsymbol{q}}\lambda }\,\exp \,(i{\boldsymbol{q}}\cdot {\boldsymbol{r}}))\end{array}$$

Here $\hat{p}=-\,i\hslash {\boldsymbol{\nabla }}$ is the momentum operator, *α* is the Rashba SOI strength, *e* is the elementary charge and $\hat{\sigma }$ is the vector of Pauli matrices. The vortex vector potential ***A***(***r***, *t*) fulfills the Lorenz gauge condition^22^ ${\boldsymbol{\nabla }}\cdot {\boldsymbol{A}}({\boldsymbol{r}},t)+$ $\mathrm{(1/}{c}^{2}){\partial }_{t}{\rm{\Phi }}({\boldsymbol{r}},t)=0$ where Φ(***r***, *t*) is the electric scalar potential that has to be included in the considerations for tightly focused optical vortices. This particular type of spatio-temporally inhomogeneous light-matter interaction is crucial for the emergence of a photo-induced unidirectional spin current. Besides, we will be dealing with short pulses accounting for all multiphoton processes which allow the generation of a substantial spin-current carrying state population.

The interaction between the non-equilibrium electrons and phonons is described by ${\hat{H}}_{{\rm{ph}}}$^60–63^ where *M*_*λ*_(***q***) is the scattering matrix element, ***q*** is the phonon wave vector, ${b}_{{\boldsymbol{q}}\lambda }^{\dagger }$ and *b*_***q****λ*_ the phonon annihilation and creation operators, respectively. The parameter *λ* denotes the polarization index. The environmental phonon bath is governed by the Hamiltonian ${\hat{H}}_{{\rm{bath}}}$ in thermal equilibrium with the occupation being characterized by the Bose-Einstein distribution function *n*(***q***) = *n*^0^(*q*). Since we are interested in low-lying excitations near the Fermi level we account for interactions with acoustic-phonons only^60,63^, while the excitation energy of optical phonons is above 30 meV^64^. In the considered InGaAs-InAs-InAlAs semiconductor quantum ring, an excited electron interacts with the longitudinal acoustic (LA) phonon modes through a deformation potential and with both the longitudinal and the transverse acoustic (TA) phonon modes through piezoelectric fields^62–66^. Therefore, the total scattering matrix element is ${|M({\boldsymbol{q}})|}^{2}={|{M}_{{\rm{LA}}}^{{\rm{DF}}}({\boldsymbol{q}})|}^{2}+{\sum }_{\lambda ={\rm{LA}},{\rm{TA}}}{|{M}_{\lambda }^{{\rm{PZ}}}({\boldsymbol{q}})|}^{2}$.

The electron-phonon interaction is spin independent, affecting the spin however when assisted by the SOI. Therefore, the treatment involves both orbital and spin relaxation due to the interaction with phonons. By orbital relaxation, we mean the transition from the excited orbital states (around the Fermi level *E*_*F*_) to all lower states. The spin relaxation is a result of the coupling of the electron spin to the phonons due to SOI and depends on the non-equilibrium level splitting through the action of the optical vortex on the spin states, characterized by the term $\frac{e\alpha }{\hslash }{[\hat{\sigma }\times {\boldsymbol{A}}({\boldsymbol{r}},t)]}_{z}$. Usually, the orbital relaxation is much faster than the spin decay which is in the range of *μ*s or longer^67–70^.

In the case of pure Rashba coupling and confinement potential with circular symmetry, the equilibrium Hamiltonian ${\hat{H}}_{0}$ commutes with the total angular momentum operator and its *z*-projection ${\hat{J}}_{z}={\hat{L}}_{z}+{\hat{S}}_{z}$ where ${\hat{S}}_{z}=\frac{\hslash }{2}{\sigma }_{z}$ is the operator of the *z* component of the spin and $\hat{L}=-\,i\hslash ({\boldsymbol{r}}\times {\boldsymbol{\nabla }})$ is the operator of the orbital angular momentum. The form of ${\hat{J}}_{z}$ reveals that its eigenvalues *j*_*z*_ are doubly degenerete, because an eigenvalue $\ell$ of ${\hat{L}}_{z}$ and 1/2 of ${\hat{S}}_{z}$ leads to ${j}_{z}=\ell +1/2$ which gives the same result as the eigenvalue pair $\ell +1$ and −1/2 of the respective operators.

To obtain the ground state spectrum we use the following basis wave functions

3 $${\varphi }_{{n}_{\uparrow },{n}_{\downarrow },\ell }({\boldsymbol{r}})={R}_{{n}_{\uparrow },\ell }(r){e}^{i\ell \phi }|\,\uparrow \,\rangle +{R}_{{n}_{\downarrow },\ell +1}(r){e}^{i(\ell +\mathrm{1)}\phi }|\,\downarrow \,\rangle $$

where $\ell$ is the angular momentum quantum number and the radial wave functions satisfy the stationary Schrödinger equation $[\frac{{\hat{p}}^{2}}{2{m}^{\ast }}+V(r)]{R}_{n,\ell }(r)={E}_{n,\ell }^{0}{R}_{n,\ell }(r)$. The radial quantum number *n* specifies the radial band. In the case of the used confinement potential $V(r)={a}_{1}/{r}^{2}+{a}_{2}{r}^{2}-2\sqrt{{a}_{1}{a}_{2}}$ ^71^ the radial states can be found analytically while the energy dispersion is given by ${E}_{n,\ell }^{0}=(n+\frac{1}{2}+\frac{1}{2}\sqrt{{\ell }^{2}+\frac{2{m}^{\ast }{a}_{1}}{{\hslash }^{2}}})\hslash {\omega }_{0}-\frac{{m}^{\ast }}{4}{\omega }_{0}^{2}{r}_{0}^{2}$ where ${\omega }_{0}=\sqrt{8{a}_{2}/{m}^{\ast }}$ and *r*_0_ = (*a*_1_/*a*_2_)^1/4^. The usage of the basis defined in Eq. (3) is advantageous when calculating the matrix elements $\langle {\varphi }_{i}|{\hat{H}}_{0}|{\varphi }_{j}\rangle$ (we used the shorthand notation $i=\{{n}_{\uparrow },{n}_{\downarrow },\ell \}$ and separated the radial and angular degrees of freedom)^72–74^. The resulting matrix is block-diagonal where every individual block corresponds to a specific (orbital) angular quantum number $\ell$ and has the size 2(*n*_max_ + 1) × 2(*n*_max_ + 1) where *n*_max_ is the number of considered radial bands. Therefore, every block can be diagonalized individually. Finally, the resulting equilibrium electron states can be expressed as

4 $${{\rm{\Psi }}}_{n,j}^{(\pm )}({\boldsymbol{r}})=\sum _{{n}_{\uparrow }=0}^{{n}_{max}}\,{a}_{{n}_{\uparrow },\ell }^{n,(\pm )}{R}_{{n}_{\uparrow },\ell }(r){e}^{i\ell \phi }|\,\uparrow \,\rangle +\sum _{{n}_{\downarrow }=0}^{{n}_{max}}\,{b}_{{n}_{\downarrow },\ell }^{n,(\pm )}{R}_{{n}_{\downarrow },\ell +1}(r){e}^{i(\ell +1)\phi }|\,\downarrow \,\rangle .$$

**Figure 2:**
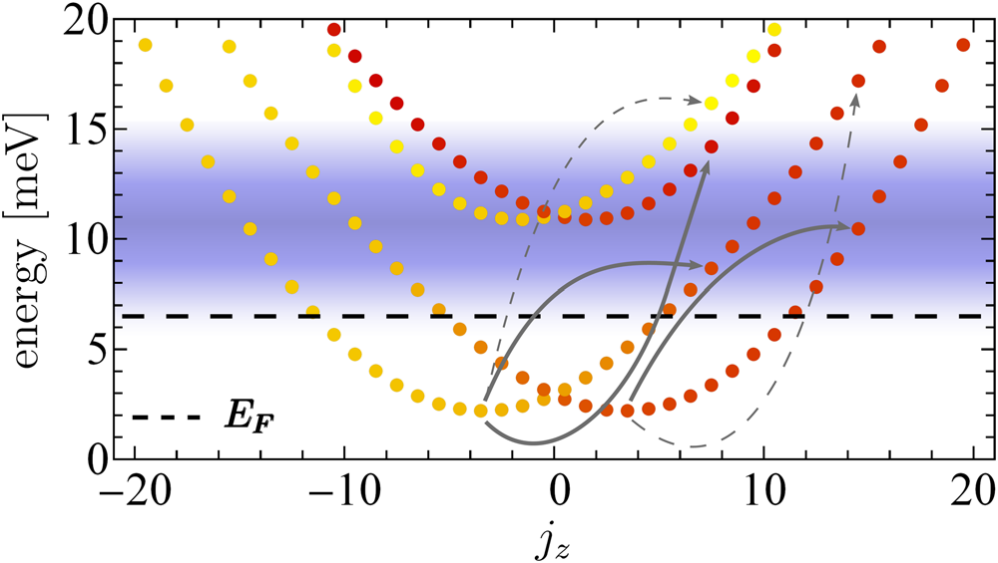
Stationary energy spectrum of the quantum ring with Rashba SOI. The states are labeled by the eigenvalues of the *z* component of the total angular momentum ${j}_{z}={\ell }_{z}+{s}_{z}$ (color gradient indicates from yellow for $\langle {\sigma }_{z}\rangle  < 0$ through red for $\langle {\sigma }_{z}\rangle  > 0$). *E*_*F*_ is Fermi level. The blue horizontal bar marks the spectral width of the four-cycle optical vortex pulse with photon energy *ħω*_*x*_ = 8.5 meV and topological charge *m*_OAM_ = 12, excitations beyond this region are also possible via multiphoton processes. The thick grey arrows indicate probable transitions pathways for moderately intense laser while the dashed arrows highlight excitations with very low probability. The radial potential of the quantum ring with a radius *r*_0_ = 50 nm has a confinement strength *ħω*_0_ = 8.5 meV while the SOI strength is *α* = 30 meV nm corresponding to *ħω*_*R*_ = 2 *α*/*r*_0_ = 1.2 meV.

A typical energy spectrum for a quantum ring with Rashba SOI is shown in Fig. 2. It reveals doubled parabolic-like energy curves representing different radial modes *n* = 0, 1, 2 which also characterizes the number of nodes of the corresponding wave functions. The two states corresponding to the same radial mode *n* and value ${j}_{z}=\ell +{s}_{z}$ are orthogonal if we take the spin degree of freedom into account. The resulting eigenenergies and states of ${\hat{H}}_{0}$ can be labeled as ${E}_{n,{j}_{z}}^{(\pm )}$, $|{{\rm{\Psi }}}_{n,{j}_{z}}^{(\pm )}\rangle$ respectively, with *j*_*z*_ and *n* referring to the spatial degrees of freedom (azimuthal and radial coordinates) while the labels (+) and (−) distinguish between positive ($\langle {{\rm{\Psi }}}_{n,j}^{(+)}|{\sigma }_{z}|{{\rm{\Psi }}}_{n,j}^{(+)}\rangle  > 0$) and negative spin orientation ($\langle {{\rm{\Psi }}}_{n,j}^{(-)}|{\sigma }_{z}|{{\rm{\Psi }}}_{n,j}^{(-)}\rangle  < 0$).

The vector potential of a linearly polarized Laguerre-Gaussian vortex beam in the plane *z* = 0 is given by ${\boldsymbol{A}}({\boldsymbol{r}},t)=\hat{\varepsilon }{A}_{0}{f}_{{m}_{{\rm{OAM}}}}(r)\,{\rm{\Omega }}(t)\,\exp [i({m}_{{\rm{OAM}}}\phi -{\omega }_{x}t)]+{\rm{c}}{\rm{.c}}$ where *A*_0_ is the amplitude, $\hat{\varepsilon }={\hat{\varepsilon }}_{x}$ the polarization vector, ${\rm{\Omega }}(t)={\cos }^{2}(\pi t/{T}_{p})$ the pulse envelope for $t\in [\,-\,{T}_{p}\mathrm{/2},{T}_{p}\mathrm{/2]}$, ${T}_{p}=2\pi {n}_{{\rm{cyc}}}/{\omega }_{x}$ the pulse length, *n*_cyc_ the number of optical cycles. This type of optical vortices characterizes a diffractive light wave: indeed, the primary propagation direction (and therefore the direction of the linear momentum) is in the *z*-direction. However, it consists also of plane waves with a distribution of different transverse momenta with the consequence that ${\boldsymbol{\nabla }}\cdot {\boldsymbol{A}}({\boldsymbol{r}},t)\ne 0$. An important quantity is the topological number *m*_OAM_ which characterizes the longitudinal component of the (carried) orbital part of the angular momentum as an intrinsic property of the optical vortex^75^. Optical vortices reveal a transverse extrinsic orbital part of the angular momentum which is the origin of the spin Hall effect of light (transverse beam shift) in optical reflections^76^. We note that the separation in the spin and orbital part of the angular momentum is generally not gauge invariant while the *total* angular momentum is invariant^77,78^. The spin and orbital parts of the angular momentum correspond to distinct symmetries of the free electromagnetic field and hence are separately conserved quantities^79^. In the Lorenz gauge, the total (longitudinal) orbital and spin angular momenta $\ell \hslash$ and *σ*_*z*_*ħ* for the Laguerre-Gaussian modes are obtained. The considered radial distribution function of the Laguerre-Gaussian mode^22^ has a radial index *p* = 0, i.e. ${f}_{{m}_{{\rm{OAM}}}}(r)={(\sqrt{2}r/{w}_{0})}^{{m}_{{\rm{OAM}}}}\,\exp (\,-\,{r}^{2}/{w}_{0}^{2})$. Note, that the usual normalization constant is incorporated into the laser amplitude *A*_0_ and we used that the generalized Laguerre polynomials ${L}_{p=0}^{{m}_{{\rm{OAM}}}}(x)=1$. Using the Lorenz gauge the electric scalar potential is given by ${\rm{\Phi }}({\boldsymbol{r}},t)=-\,{c}^{2}{\int }_{-\infty }^{t}\,{\rm{d}}t^{\prime} \,{\boldsymbol{\nabla }}\cdot {\boldsymbol{A}}({\boldsymbol{r}},t^{\prime} )$. The intensity profile of the optical vortex beam forms a donut shape focused onto the ring.

The interaction of the charge carriers with the optical vortex beam leads to an imbalance in the internal OAM state with an amount determined by the optical topological charge *m*_OAM_: ${\ell }_{f}={\ell }_{i}+{m}_{{\rm{OAM}}}\pm \,1$ ^53^.

The initial degeneracy with respect to clockwise and anti-clockwise angular motion is then broken. The result is the emergence of unidirectional circulating currents (for more details we refer to the supplementary materials).

### Numerical results

We applied our theory to an InGaAs-InAs-InAlAs ring with a radius of *r*_0_ = 50 nm with a radial confinement strength *ħω*_0_ = 8.5 meV. The ring width is Δ*r* = 25 nm. The effective mass of the host material is m^*^ = 0.023*m*_*e*_ while the effective Rashba SOI strength is tunable between *α* = 5 meV nm and *α* = 40 meV nm. The central photon energy of the applied four-cylce optical vortex pulse is *ħω*_*x*_ = 8.5 meV causing transitions to the second radial band (cf. Fig. 2) for the appropriate topological charges *m*_OAM_. For a qualitative comparison between different winding numbers, the amplitude of the vector potential ***A***(***r***, *t*) has to be normalized to the photon number which will be fixed in the following discussions. The topological charge of the optical vortex applied here ranges from *m*_OAM_ = −20 to *m*_OAM_ = +20 which is possible with recent experimental techniques^30,33^. Typically, we consider moderate peak intensities around *I*_*x*_ = 10^7^ W/cm^2^.

**Figure 3:**
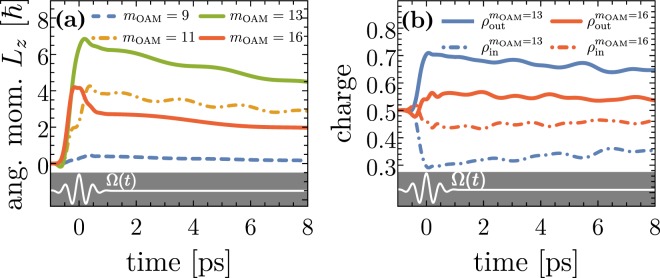
Spin-unresolved charge dynamics. (**a**) The time-dependent expectation value of the orbital angular momentum operator ${\hat{L}}_{z}$ (referring to the total quantum ring) for different topological charges *m*_OAM_ of the irradiated optical vortex beam (the black curve is temporal envelope). The values of *L*_*z*_ reflect the nicely the band width of the electromagnetic pulse. (**b**) The time-dependent charge accumulation *ρ*_in_(*t*) and *ρ*_out_(*t*) for the inner region (*r* < *r*_0_) and outer region (*r* > 0) of the quantum ring for two topological charges. Particle conservation is guaranteed since *ρ*_in_(*t*) + *ρ*_out_(*t*) ≡ 1. The black curve illustrate the temporal behaviour of the four-cycle optical vortex beam. The SOI strength is *α* = 30 meV nm.

Figure 3 shows the general influence of the optical vortex beam imparting orbital angular momentum to the charge carriers with respect to the *z* axis. Even for single-photon processes a higher topological charge *m*_OAM_ leads in general to a larger OAM change ${\rm{\Delta }}\ell ={m}_{{\rm{OAM}}}\pm 1$ as long as the transition frequency *ω*_*if*_ = (*E*_*f*_ − *E*_*i*_)/*ħ* lies within the band width of the applied pulse which is evidenced by the optical transition amplitudes (see supplementary materials). Therefore, we find a natural limitation of the maximal transferable OAM to the system given by the pulse length *T*_*p*_ which determines the band width. For clarity, in Fig. 2 the band width of the applied four-cycle optical vortex pulse is shown which also nicely explains which states are involved in the dynamics corresponding to Fig. 3(a). Here, the dynamical buildup of the *z*-component of the orbital angular momentum *L*_*z*_ of the whole quantum ring is demonstrated which is directly related to the longitudinal component of the light’s orbital angular momentum, characterized by *m*_OAM_. Note, that due to the restriction of the charge dynamics to the *x* − *y* plane the in plane components *L*_*r*_ and *L*_*φ*_ are zero. We find the highest OAM transfer in the case *m*_OAM_ = 13 because the associated transition paths belong to the position of the energetic maximum within the band width while, for instance, the case *m*_OAM_ = 16 belongs to a drastically reduced photoexcitation probability. The imparted OAM affects the spin channels via SOI. The excited and non-equilibrium spin-dependent populations interact with the phonon bath leading to irreversible relaxation processes. We find the unidirectional currents are sizable for some picoseconds. A subsequent vortex pulse is needed to build up the current again.

In Fig. 3(b) the charge accumulation at the ring boundaries is demonstrated which correlates with the gain in OAM in time^53^. Physically, the increase in the angular momentum results in a higher effective centrifugal potential pressing the charge density to the outer ring boundaries, i.e. ${\rho }_{{\rm{out}}}(t) > {\rho }_{{\rm{in}}}(t)$, provided that the quantum confinement is strong enough to avoid a spill out. Here, ${\rho }_{{\rm{in}}/{\rm{out}}}(t)={\int }_{r\lessgtr {r}_{0}}{\rm{d}}{\boldsymbol{r}}\langle {\boldsymbol{r}}|\rho (t)|{\boldsymbol{r}}\rangle$ where the radial integration is restricted to the domains *r* < *r*_0_ (*ρ*_in_) and *r* > *r*_0_ (*ρ*_out_). From Fig. 3(b) one can deduce that the strength of this non-equilibrium charge separation is proportional to the amount of the gained OAM during the interaction with the vortex beam. Note that this charge accumulation conserves the particle number since *ρ*_in_(*t*) + *ρ*_out_(*t*) ≡ 1. The pronounced charge separation decays in time due to relaxation processes as a result of the interaction between the photoexcited electron carriers and the phonon bath.

**Figure 4:**
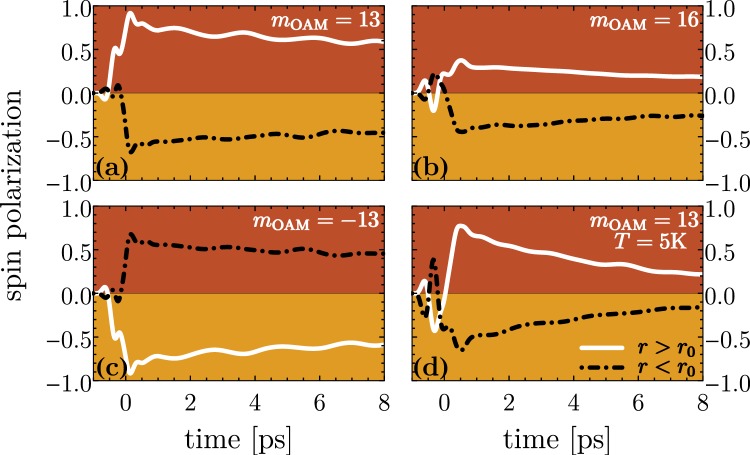
The time-dependent spin polarization of the inner (*r* < *r*_0_) and outer (*r* > *r*_0_) region of the driven quantum ring for the topological charges (**a**) *m*_OAM_ = 13, (**b**) *m*_OAM_ = 16 (**c**) *m*_OAM_ = −13 and (**d**) *m*_OAM_ = 13. The pulse and system parameters are the same as in Fig. 3. The temperatures for (**a**–**c**) is 1 K and for (**d**) is 5 K.

Recalling Lorentz reciprocity theorem, one may wonder whether the demonstrated process is reversible, meaning whether a generation of radiation carrying OAM is possible by a spin dependent loop current. At first sight one may anticipate a positive answer, at least in our case, based on the fact that the charge current in our case is a non-diffusive current (before the phonon path becomes active) and the dynamics should be indeed reversible. Apart from complications related to the spin dynamics reversibility in the presence of spin-orbital coupling, the issue of whether optical vortices (even if generated locally) can then propagate to the far region is not yet settled. In this connections, we refer to a recent study on the high harmonic emission of current carrying quantum rings^80^. We also note that an electron in circular motion may emit radiation with helical phase structure hinting on a carried OAM^81^. Along with the photoinduced unidirectional current and charge centrifugal accumulation one may expect some edge spin density accumulations. One note however that the time scale for the charge and spin dynamics are different and it is not clear on which time scale a spin current and spin accumulation will build up and persist. To gain insight, we consider the time-dependent spin polarization of the inner and outer ring regions as defined by

5 $${S}_{{\rm{in}}/{\rm{out}}}(t)=\frac{{\rho }_{{\rm{in}}/{\rm{out}}}^{\uparrow }(t)-{\rho }_{{\rm{in}}/{\rm{out}}}^{\downarrow }(t)}{{\rho }_{{\rm{in}}/{\rm{out}}}(t)}$$

where ${\rho }_{{\rm{in}}/{\rm{out}}}^{\uparrow /\downarrow }(t)={\int }_{r\lessgtr {r}_{0}}\,{\rm{d}}{\boldsymbol{r}}\langle {\boldsymbol{r}}|{\rm{T}}r\{{{\rm{\Lambda }}}_{\uparrow /\downarrow }\rho (t)\}|{\boldsymbol{r}}\rangle$ and ${{\rm{\Lambda }}}_{\uparrow /\downarrow }$ is the projection operator on the spin-up and spin down states of *σ*_*z*_, respectively. Figure 4 presents the spin polarization of the inner end exterior ring regions for two different optical vortex beam setups. Both regions are initially spin unpolarized, meaning *S*_in/out_(*t* → −∞) = 0 in equilibrium. During the laser interaction the increasing spin separation in the non-equilibrium state builds up. While the charge carriers at the outer ring boundary show more and more a definite positive spin polarization, we find an accumulation of spin-down states at the inner barrier in time implying a photo-induced spin Hall effect. Note, |*S*_in_| ≠ |*S*_out_| which reflects the fact that ${[\hat{H}(t),{\sigma }_{z}]}_{-}\ne 0$ (cf. also Fig. 2). For a positive topological charge an initial state in the spin-up channel (+) ($\langle {\sigma }_{z}\rangle  > 0$, red dots in Fig. 2) undergoes a transition (while changing the internal angular momentum by *m*_OAM_) to a spin up-channel in the second radial band. In the single photon regime, a spin-flip transition (to a yellow state) is not favored because the corresponding frequency is not within the pulse band width. For a high angular momentum transfer, the charge density is repelled to the outer ring boundary effectively spin-polarizing the exterior region.

In contrast, for a state in the spin-down channel ($\langle {\sigma }_{z}\rangle  > 0$), yellow dot in Fig. 2), a direct spin-flip transition is possible and governed by ${\hat{H}}_{{\rm{S}}{\rm{O}}{\rm{I}}}(t)=\frac{\alpha }{\hslash }{[\hat{H}\times {\boldsymbol{A}}({\boldsymbol{r}},t)]}_{z}$ as part of ${\hat{H}}_{{\rm{int}}}(t)$ (see supplementary materials) since the corresponding transition frequency is within the band width of the applied beam. Thus, starting with a spin-down state (yellow dot), the pulse generates a mixture of spin-flip and spin-conserving transitions. Concluding, we predict the emergence of optical vortex-induced spin Hall drift current due to the transfer of OAM, residual SOI, confinement effects (dictating the initial populations), and temporal properties of the optical vortex beam.

The same mechanism applies when changing the sign of the topological charge leading to a switch in the direction of the spin Hall current, meaning the photo-induced current loop proceeds in the opposite direction leading to negative spin polarization of the exterior domain and an accumulation of spin-up electronic states at the inner boundary, shown in Fig. 4(c). Just like the charge accumulation, depicted in Fig. 3(b), also the spin separation depends strongly on the amount of the carried and transferred total OAM to the quantum ring system. In Fig. 4(a), the situation for *m*_OAM_ = 13 is shown which we identify from Fig. 3(a) as the laser beam setup where the nano-structure gains the maximal amount of OAM. Here, we find a strong spin polarization of the inner and outer regions. Around 80 percent spin polarization at the outer ring boundary can be achieved on a ps-time scale just before relaxation becomes sizable. A slight increase of the topological charge to *m*_OAM_ = 16 (panel 4(b)) results in a distinctly lowered spin separation which comes along with the reduced OAM transfer (cf. Figure 3(a)) to the system. The effect of temperatures is illustrated by panel (d) of Fig. 4 that shows the evolution of *S*_in/out_ for *T* = 5 K. Due to the chosen weak confinement and small *E*_*F*_ the initial electronic population is substantially altered and, hence, we find a relatively strong influence of temperatures. However, the general concept does not rely on a specific initial distribution. Rather, it is the residual SOI that in effect converts the optical orbital angular momentum into directed drift spin current.

**Figure 5:**
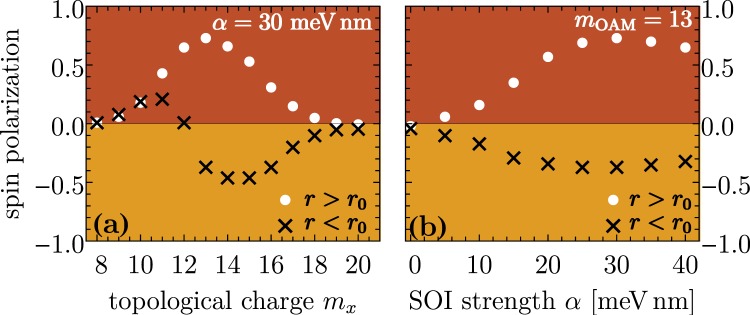
(**a**) The dependence of the spin polarization of the interior and exterior ring regions on the topological charge *m*_OAM_ of the applied optical vortex pulse. (**b**) Spin polarizations for different Rashba SOI strengths *α*. The other laser and system parameters are the same as those in Fig. 2.

Figure 5(a) summarizes the dependence on the topological charge of the applied optical vortex beam. For this purpose, let us introduce the time interval *T*_obs_ straight after the laser pulse which is chosen in a way that the effective relaxation mechanism is not too pronounced meaning *T*_obs_ < *τ*_rel_. Here, *τ*_rel_ is the effective relaxation time which is around 25 ps as revealed by the Redfield tensor in Eq. (9). A reasonable choice is *T*_obs_ = 2 ps. As a consequence, the average spin polarizations

6 $${\overline{S}}_{{\rm{In}}/{\rm{Out}}}=\frac{1}{{T}_{{\rm{obs}}}}{\int }_{{T}_{p}\mathrm{/2}}^{{T}_{p}\mathrm{/2}+{T}_{{\rm{obs}}}}{S}_{{\rm{In}}/{\rm{Out}}}(t).$$

are characteristic quantities for the non-equilibrium time regime. As expected we find a maximal spin separation and most pronounced spin polarization in the case *m*_OAM_ = 13. For topological charges below *m*_OAM_ = 8 or higher than *m*_OAM_ = 19, we find no spin separation due to a vanishing transfer of OAM because the corresponding transition frequencies are beyond the pulse band width, i.e., higher non-linear processes turned out to be very small. An interesting behaviour shows the spin polarization of the inner region which undertakes a transition from a positive spin orientation for smaller *m*_OAM_ to a spin-down polarization for larger *m*_OAM_. Thus, the pulse band width together with the amount of transferred OAM (determined by *m*_OAM_) determines whether spin-conserving or spin-flip transitions occur.

Figure 5(b) demonstrate the role of the Rashba SOI strength *α* for a topological charge *m*_OAM_ = 13. Obviously, no spin separation occurs for *α* → 0. In the domain *α* ∈ [0, 30 meV nm], the spin separation (polarization) enhances with increasing the Rashba SOI strength. Surprisingly, for coupling strengths above *α* = 30 meV nm the spin separation starts decreasing. One can explain this phenomenon with spin crossing effects. Our equilibrium calculations of the electronic structure, depicted in Fig. 2, reveal that for *α* > 20 meV nm the spin channels are strongly mixed. Thus, optically induced transitions are accompanied with spin-flips leading so to damping of the overall spin polarization.

A key quantity describing SHE is the spin Hall angle (SHA) given as the ratio between the transversal spin current and the longitudinal charge current for moderate bias. For a dc electric field in the steady state, the SHA is defined in the linear response regime as ${\alpha }_{{\rm{SHA}}}={\sigma }_{xy}^{s}/{\sigma }_{xx}$, meaning the ratio of the spin Hall conductivity ${\sigma }_{xy}^{s}$ and the diagonal term *σ*_*xx*_ of the conductivity tensor^18^. In our case, we deal with time-dependent non-linear processes which are captured through the numerical propagation of the density matrix. For a meaningful defintion of SHA in this regime, we use the spin-resolved current density ${{\boldsymbol{j}}}^{s}({\boldsymbol{r}},t)={\rm{T}}r\{{{\rm{\Lambda }}}_{s}\hat{\rho }(t)\hat{{\boldsymbol{j}}}\}$. The operator $\hat{{\boldsymbol{j}}}({\boldsymbol{r}},t)=\frac{e}{2{m}^{\ast }}[|{\boldsymbol{r}}\rangle \langle {\boldsymbol{r}}|\hat{{\boldsymbol{\pi }}}+{\hat{{\boldsymbol{\pi }}}}^{\dagger }|{\boldsymbol{r}}\rangle \langle {\boldsymbol{r}}|]$ with $\hat{{\boldsymbol{\pi }}}=\hat{{\boldsymbol{p}}}-e{\boldsymbol{A}}({\boldsymbol{r}},t)$ and Λ_*s*_ projects on the eigenfunctions |*s* = ↑, ↓〉 of ${\hat{\sigma }}_{z}$. The dynamical, non-linear SHA we quantify by

7 $${\alpha }_{{\rm{SHA}}}(t)=\frac{{I}_{r}^{\uparrow }(t)-{I}_{r}^{\downarrow }(t)}{{I}_{\phi }^{\uparrow }(t)+{I}_{\phi }^{\downarrow }(t)}$$

where ${{\boldsymbol{I}}}^{s}(t)=\int {\rm{d}}{\boldsymbol{r}}\,{{\boldsymbol{j}}}^{s}({\boldsymbol{r}},t)$. Thus, *α*_SHA_(*t*) describes the ratio between the transverse *spin-polarized* current ${I}_{r}^{\uparrow }(t)-{I}_{r}^{\downarrow }(t)$ (cf. Fig. 4) and the angular charge current in ${\hat{\varepsilon }}_{\phi }$.

**Figure 6:**
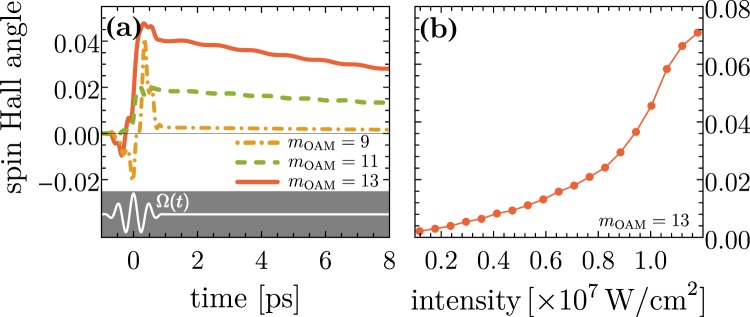
(**a**) Buildup of dynamical spin Hall angle (SHA) for different topological charges *m*_OAM_ at fixed intensity. An optically-induced orbital angular momentum leads to a higher (photo-induced) charge current and stronger effective spin-orbital effects (cf. Fig. 4). (**b**) Dynamical SHA vs. laser peak intensity. Linear response is valid for intensities below 5 × 10^6^ W/cm^2^.

In Fig. 6(a) the general temperoral characteristics and dependence on the topological charge of the applied optical vortex are shown. The pulse parameters are the same as those used for the results depicted in Figs 3–5. An increase of the winding number leads to a higher current effectively enlarging the SHA as long as the transition frequency *ω*_*if*_ is in the band width of the four-cycle pulse. In the case of *m*_OAM_ = 13 we find the largest SHA which corresponds to the largest acquired angular momentum by the quantum ring structure (cf. Fig. 3(a)). Panel (b) depicts the influence of the laser peak intensity when averaging over the observation interval *T*_obs_ straight after the laser pulse (cf. Eq. 6). The results indicate three regimes: below 5 × 10^6^ W/cm^2^, the driven quantum ring exhibits a nearly linear-response to laser field strength. For intensities larger than 5 × 10^6^ W/cm^2^, the SHA increases rapidly signifying non-linearity. Thus, our results for *I*_*x*_ = 10^7^ W/cm^2^ (Figs 3–5) are in the non-linear regime. Increasing the intensity further, we observe a saturation effect. Current technical limitations prevent using higher peak intensities in our numerics as the needed basis set of eigenfunctions increases drastically because multi-photon process become dominant.

## Discussion

We demonstrated an efficient way for ultrafast photo-induced unidirectional spin Hall current pulses in a two-dimensional semiconductor quantum ring using a few-cycle optical vortex beam. The imparted optical orbital angular momentum ignites a unidirectional looping charge current which, due to the residual spin-orbital coupling induces a drift spin current leading to edge spin accumulation. These phenomena can be controlled on the picosecond time scale by the parameters of the pulse, for instance the magnitude of the spin current can be enhanced by increasing the optical topological charge while keeping the laser intensity unchanged making the method noninvasive. The mechanism relies on the topology of the light field OAM rather than on the system crystal symmetry and should thus be present for a wide range of structured materials with spin-orbital coupling. A large number of important applications based on the revealed phenomena can be envisaged. The triggered boundary spin accumulation is large and can be exploited as a source for an ultrafast spin transfer torque when interfaced with a ferromagnetic material. Further, when appropriately contacted with a conductor with a strong spin-orbit scattering (such as a Pt stripe) the spin density buildup generates a charge current pulse via the inverse spin Hall effect.

## Methods

In its equilibrium state, the initial density matrix of the quantum ring reads ${\rho }_{i,j}^{0}(t\to -\,\infty )={f}_{i}^{0}({E}_{F},T){\delta }_{i,j}$ where ${f}_{i}^{0}({E}_{F},T)$ is the Fermi-Dirac distribution^82^. The excitation dynamics of the system is governed by Heisenberg’s equation of motion

8 $$i\hslash {\partial }_{t}{\rho }_{m,m^{\prime} }(t)={[{\hat{H}}_{0}+{\hat{H}}_{{\rm{int}}}(t),{\rho }_{m,m^{\prime} }(t)]}_{-}-\sum _{kl}\,{R}_{mm^{\prime} kl}{\rho }_{kl}(t)$$

which is space-time-grid propagated in the presence of the vortex vector potential and includes thus non-linear effects in the external field. Irreversible open system dynamics is also captured (on the level outlined above). Initially and in equilibrium, the system described by ${\hat{H}}_{0}$ and the phonon bath ${\hat{H}}_{{\rm{b}}{\rm{a}}{\rm{t}}{\rm{h}}}$ are uncorrelated, meaning that the total density matrix is cast as ${\rho }_{{\rm{t}}{\rm{o}}{\rm{t}}}(t\to -\,{\rm{\infty }})=\rho (t\to -\,{\rm{\infty }}){\rho }_{{\rm{B}}}(t\to -\,{\rm{\infty }})$ where ${\rho }_{B}={e}^{-{\hat{H}}_{{\rm{b}}{\rm{a}}{\rm{t}}{\rm{h}}}/{k}_{B}T}/{Z}_{B}$ with ${Z}_{B}={\rm{T}}{\rm{r}}\{{e}^{-{\hat{H}}_{{\rm{b}}{\rm{a}}{\rm{t}}{\rm{h}}}/{k}_{B}T}\}$. Upon excitations the system-bath coupling sets in. We employ the following approximations: the coupling to the phonon bath is weak allowing the use of the Born approximation ${\rho }_{{\rm{tot}}}(t)\simeq \rho (t){\rho }_{B}(t\to -\,\infty )$. We assume that the time evolution of $\rho (t)$ depends only on its present value and not on its past state, meaning we use the Markov approximation. Then, the system (reduced) density matrix $\rho (t)={{\rm{Tr}}}_{B}\{{\rho }_{{\rm{tot}}}(t)\}$ in Eq. (8) traced over all degrees of freedom of the phonon reservoir *B* can be obtained^83–85^. Using standard density matrix algebra, the Redfield tensor is expressed as

9 $${R}_{nmkl}={\delta }_{nm}\sum _{r}\,{{\rm{\Gamma }}}_{nrrk}^{+}+{\delta }_{nk}\sum _{r}\,{{\rm{\Gamma }}}_{lrrk}^{-}-{{\rm{\Gamma }}}_{lmnk}^{+}-{{\rm{\Gamma }}}_{lmnk}^{-}$$

in terms of transition rates governed by Fermi golden rules expressions

10 $${{\rm{\Gamma }}}_{lmnk}^{+}={\int }_{0}^{\infty }\,{\rm{d}}t\,{e}^{-i{\omega }_{nk}t}\overline{\langle l|{\hat{H}}_{{\rm{ph}}}|m\rangle \langle n|{\hat{H}}_{{\rm{ph}}}(t)|k\rangle }.$$

Here we use that $${{\rm{\Gamma }}}_{lmnk}^{+}={({{\rm{\Gamma }}}_{knml}^{-})}^{\ast }$$, while $${\hat{H}}_{{\rm{p}}{\rm{h}}}(t)=\exp (i{\hat{H}}_{{\rm{b}}{\rm{a}}{\rm{t}}{\rm{h}}}t/\hslash ){\hat{H}}_{{\rm{p}}{\rm{h}}}\exp (\,-\,i{\hat{H}}_{{\rm{b}}{\rm{a}}{\rm{t}}{\rm{h}}}t/\hslash )$$. The overbar donates the average over the phonon bath in thermal equilibrium at a temperature *T* meaning $$\overline{\langle l|{\hat{H}}_{{\rm{ph}}}|m\rangle \langle n|{\hat{H}}_{{\rm{ph}}}(t)|k\rangle }=$$$${{\rm{Tr}}}_{B}\{\langle l|{\hat{H}}_{{\rm{ph}}}|m\rangle \langle n|{\hat{H}}_{{\rm{ph}}}(t)|k\rangle {\rho }_{B}\}$$. A detailed description of the transition rates is given in the supplementary materials.

Crucial for obtaining the transition rates ${{\rm{\Gamma }}}_{lmnk}^{+}$ is the calculation of all phonon matrix elements $\langle n|{\hat{H}}_{{\rm{ph}}}|m\rangle$ between all states shown in the spectrum in Fig. 2. Since we are using spin-resolved eigenstates (Eq. 4) of ${\hat{H}}_{0}$, charge and spin relaxation processes due to the spin admixture as a spin-orbit coupling phenomenon are included. For instance, the diagonal elements of ${R}_{mm^{\prime} kl}$ reveal the spin relaxation rate which is explicitly given by ${T}_{1,mm^{\prime} }^{-1}=2{\rm{Re}}({{\rm{\Gamma }}}_{m,m^{\prime} ,m^{\prime} ,m}^{+}+{{\rm{\Gamma }}}_{m^{\prime} ,m,m,m^{\prime} }^{+})$^84^. Note, that the off-diagonal elements of the Redfield tensor can be identified as decoherence rates due to the interaction with the reservoir and are responsible for the time decay of the optical excitations initiated by ${\hat{H}}_{{\rm{int}}}(t)$. Heisenberg’s equation of motion in Eq. 8 is solved using a numerically stable Leapfrog algorithm.

By solving Eq. (8) we neglect Coulomb-interactions between the charge carriers. There are several reasons for this doing: in the stationary case, i.e. $t\to -\,\infty$, it can be demonstrated that for a small number of electronic states in a quantum ring with no impurity the inclusion of correlation via Coulomb matrix elements simply shifts the non-interacting spectrum to higher energies^86^. Therefore, we calculated numerically various Coulomb matrix elements ${V}_{abcd}=\langle {{\rm{\Psi }}}_{a}{{\rm{\Psi }}}_{b}|V({\boldsymbol{r}}-{\boldsymbol{r}}^{\prime} )|{{\rm{\Psi }}}_{c}{{\rm{\Psi }}}_{d}\rangle$ where $V({\boldsymbol{r}}-{\boldsymbol{r}}^{\prime} )={e}^{2}/\mathrm{(4}\pi {\varepsilon }_{0}{\varepsilon }_{r}|{\boldsymbol{r}}-{\boldsymbol{r}}^{\prime} |)$ and we can confirm that they are actually smaller than the kinetic energy matrix elements. Thus, correlation effects lead only to a small perturbation of the underlying electronic spectrum. Importantly, the Coulomb matrix elements conserve the angular momentum, i.e. ${m}_{a}={m}_{c}$ and ${m}_{b}={m}_{d}$^87^. Since we consider optically induced intra (conduction)band transitions which change the internal angular momentum state, the Coulomb matrix elements between the states which are involved in the electric transition disappear. Consequently, correlation effects play a minor role for the qualitative description of the considered transitions. We made sure that the parameters of the pulses are chosen to trigger mainly excitations near the Fermi-level in which case the approximation discussed above remain credible.

## Electronic supplementary material

Supplementary materials
