# Supplementary material for: All-optical generation and ultrafast tuning of non-linear spin Hall current
Source: Sci Rep. 2018 Nov 20;8:17102. doi: 10.1038/s41598-018-35378-4 (PMC6243999; doi:10.1038/s41598-018-35378-4)
Supplement: Supplementary file 1 — Supplementary materials [file 41598_2018_35378_MOESM1_ESM.pdf]

Supplemental Materials: All-optical generation and ultrafast tuning of non-linear spin Hall current

Jonas Wätzel[1,*] **and Jamal Berakdar**[1]

[1]Institute for Physics, Martin-Luther-University Halle-Wittenberg, 06099 Halle, Germany
[*]jonas.waetzel@physik.uni-halle.de

1 Dissipative Dynamics

In the low-temperature limit, the interaction between the phonon reservoir B and the quantum ring is written as the product

$$\hat{H}_{\text{ph}} = \sum_{\boldsymbol{q}} Q_{\boldsymbol{q}} F_{\boldsymbol{q}} \tag{1}$$

where $Q_{\boldsymbol{q}} = M_\lambda(\boldsymbol{q}) \exp(-i\boldsymbol{q}\cdot\boldsymbol{r})$ is an operator acting on the ring states and $F_{\boldsymbol{q}} = b^\dagger_{\boldsymbol{q}\lambda}$ is a bath operator (we neglect phonon absorption in the low temperature limit). In this case the Redfield-tenstor components are given by[1]

$$\begin{aligned} \Gamma^{+}_{lmnk} =& \sum_{\nu,\nu'} \langle l|Q_{\boldsymbol{\nu}}|m\rangle\langle n|Q_{\boldsymbol{\nu'}}|k\rangle \\ & \times \int_0^\infty \mathrm{d}t \langle F_{\boldsymbol{\nu}} F_{\boldsymbol{\nu'}}(t)\rangle_B e^{-i\omega_{nk}t} \\ \Gamma^{-}_{lmnk} =& \sum_{\nu,\nu'} \langle l|Q_{\boldsymbol{\nu}}|m\rangle\langle n|Q_{\boldsymbol{\nu'}}|k\rangle \\ & \times \int_0^\infty \mathrm{d}t \langle F_{\boldsymbol{\nu}}(t) F_{\boldsymbol{\nu'}}(0)\rangle_B e^{-i\omega_{lm}t}. \end{aligned} \tag{2}$$

The bath-dependent factors can be expressed by the bath correlation function $\langle F_{\boldsymbol{\nu}} F_{\boldsymbol{\nu'}}(t)\rangle_B = \mathrm{Tr}\left\{F_{\boldsymbol{\nu}} F_{\boldsymbol{\nu'}}(t)\rho_B\right\}$ and $\langle F_{\boldsymbol{\nu}}(t) F_{\boldsymbol{\nu'}}\rangle_B = \mathrm{Tr}\left\{F_{\boldsymbol{\nu}}(t) F_{\boldsymbol{\nu'}}\rho_B\right\}$. A striking property is the decay to zero within a certain time scale, which is set by the bath parameter dependent correlation time t_c. Here, the bath time-correlation function is given by[2]

$$\langle F(0)F(t)\rangle_B = \frac{1}{\pi}\int_0^\infty \mathrm{d}\omega J(\omega)\left[e^{-i\omega t} + 2n^0(\omega)\cos(\omega t)\right]. \tag{3}$$

Here, $n^0(\omega)$ is the Bose-Einstein distribution and

$$J(\omega) = \frac{\pi}{2}\sum_{\boldsymbol{q}} |a_{\boldsymbol{q}}|^2 \delta(\omega - \omega_{\boldsymbol{q}}) \tag{4}$$

is the bath spectral density of the Boson modes $\boldsymbol{q}$. The connection to the system is characterized by the weights $|a_{\boldsymbol{q}}|^2$ (coupling strength to the electron charges) which are related to the operator $Q_{\boldsymbol{q}}$ by[3,4]:

$$a_{\boldsymbol{q}} = f(\boldsymbol{q})M(\boldsymbol{q}), \tag{5}$$

where $M(\boldsymbol{q})$ is the scattering matrix element (for the acoustic phonons) and $f(\boldsymbol{q}) = \int \mathrm{d}\boldsymbol{r} n_e(\boldsymbol{r}) e^{-i\boldsymbol{q}\cdot\boldsymbol{r}}$ is the form factor depending on the (equilibrium) charge density distribution $n_e(\boldsymbol{r}) = \sum_i^{\mathrm{occ.}} |\Psi_i(\boldsymbol{r}, t \to -\infty)|^2$ in the ring.
Assuming linear acoustic phonons with a dispersion $\omega_{\boldsymbol{q}} = s|\boldsymbol{q}|$ where s is the sound velocity, we consider only the piezoelectric phonons and neglect contribution from the deformation potential which is justifiable at temperatures below 10 K for bulk GaAs or InAs material[3,5]. Therefore, $M(\boldsymbol{q})^2 = g_{\mathrm{pz}} \pi^2 s^2 / (V|\boldsymbol{q}|)$ where V is the volume of the unit cell and g_{pz} the dimensionless piezoelectric constant ($g_{\mathrm{pz}} = 0.45$ for InAs[6]). We evaluated the spectral density function $J(\omega)$ numerically by transforming the sum in Eq. (4) into an integral. By fitting we found that the high-frequency tail falls off like $J(\omega \to \infty) \propto 1/\omega$ (Ohmic behavior). The bath correlation function is obtained from Eq. (3) and a subsequent (numerically performed) Fourier transform leads to the bath-dependent parts of the Redfield components in Eq. (2) where we also need the scattering matrix elements $\langle i|\hat{Q}_{\boldsymbol{\nu}}|j\rangle$ of the electron-phonon interaction between the various spin-resolved electronic states of the quantum ring.

2 Dynamics by optical vortex pulse

The typical time scale of the acoustic phonon-induced relaxation processes in III-V semiconductor nanostructures is in the range of $> 10\,\mathrm{ps}$[7], much longer than the considered few-cycle vortex pulse length T_p . Therefore, during the action of the pulse, the relaxation is marginal.
An equilibrium electronic state characterized by the wave function $\Psi_i(\boldsymbol{r})$ and the energy E_i (note that we used the shortened notation $i = \{n, j, (\pm)\}$) can be decomposed in

$$\Psi_{n,j}^{(\pm)}(\boldsymbol{r}) = \sum_{n_\uparrow=0}^{n_{\max}} a_{n_\uparrow,\ell}^{n,(\pm)} |n_\uparrow, \ell\rangle |\uparrow\rangle + \sum_{n_\downarrow=0}^{n_{\max}} b_{n_\downarrow,\ell}^{n,(\pm)} |n_\downarrow, \ell+1\rangle |\downarrow\rangle, \tag{6}$$

where the (orbital) sub-states are given by $\langle \boldsymbol{r}|n,\ell\rangle = R_{n,\ell}(r) e^{i\ell\varphi}$ with the radial wave functions $R_{n,\ell}(r)$ and angular quantum number ℓ (φ is the polar angle).
In the presence of the optical vortex, the various (sub)states $|n,\ell\rangle|s\rangle$ are coupled due to different mechanisms. First, the direct (light-matter) interaction as part of $\hat{H}_{\mathrm{int}}$ with the optical vortex pulse is given by $\hat{H}_D = \frac{e}{2m^*}[\hat{\boldsymbol{p}} \cdot \boldsymbol{A}(\boldsymbol{r},t) + \boldsymbol{A}(\boldsymbol{r},t) \cdot \hat{\boldsymbol{p}}]$ and acts on the orbital part of the electron state. Thus, this Hamiltonian leaves the spin orientation $|s\rangle$ corresponding to the sub-states $|n,\ell\rangle$ (with coefficients $a_{n_\uparrow,\ell}^{n,(\pm)}$ and $b_{n_\downarrow,\ell}^{n,(\pm)}$) unaffected. The orbital coupling of the (orbital) sub-states is characterized by the (transition) matrix elements $\mathcal{M}_{n_j,n_i}^{\ell_j,\ell_i} = \langle n_j \ell_j|\hat{H}_D|n_i \ell_i\rangle$ which read explicitly

$$\begin{aligned} \mathcal{M}_{n_j,n_i}^{\ell_j,\ell_i} = & \frac{ieA_0\hbar}{m_e} \sum_{\lambda=\pm 1} \left[\frac{2}{w_0^3} u_{n_j,n_i}^{\ell_j,\ell_i+\lambda} \right. \\ & - \frac{1}{w_0} \left(m_{\mathrm{OAM}}(1+\lambda) + 2\lambda\ell_i - 1 \right) v_{n_j,n_i}^{\ell_j,\ell_i+\lambda} \\ & \left. - \frac{2}{w_0} w_{n_j,n_i}^{\ell_j,\ell_i+\lambda} \right] \delta_{\ell_j-\ell_i, m_{\mathrm{OAM}}+\lambda}. \end{aligned} \tag{7}$$

The summation over λ stems from the linear polarization of the optical vortex in the x-direction. The $\delta_{n,n'}$ is the Kronecker symbol while the coefficients u, v and w are radial integrals (in polar

coordinates):

$$
\begin{aligned}
u_{n',n}^{\ell',\ell} &= \int_0^\infty \mathrm{d}r\, r R_{n',\ell'}(r) R_{n,\ell}(r) f_{m_{\mathrm{OAM}}}(r), \\
v_{n',n}^{\ell',\ell} &= \int_0^\infty \mathrm{d}r\, R_{n',\ell'}(r) R_{n,\ell}(r) f_{m_{\mathrm{OAM}}}(r)/r \quad \text{and} \\
w_{n',n}^{\ell',\ell} &= \int_0^\infty \mathrm{d}r\, R_{n',\ell'}(r) \partial_r [R_{n,\ell}(r)] f_{m_{\mathrm{OAM}}}(r).
\end{aligned} \tag{8}
$$

Note that $f_{m_{\mathrm{OAM}}}(r)$ is the radial distribution function of the optical vortex beam which dependents on the topological charge m_{OAM}. Noticeably, the light-matter interaction leads to transitions from $|\Psi_n\rangle$ to quantum states $|\Psi_{n'}\rangle$ with higher orbital angular momenta as dictated by the winding number m_{OAM}. Interestingly, although $\hat{H}_{\mathrm{D}}(t)$ does not act directly on the spin-orientation of the sub-states, a spin-flip transition is nevertheless possible due to the orbital coupling between sub-states of the two different (spin) channels $(+)$ and $(-)$.

The SOI interaction $\hat{H}_{\mathrm{SOI}}(t) = \frac{\alpha}{\hbar}\left[\hat{\sigma} \times \boldsymbol{A}(\boldsymbol{r},t)\right]_z$ describes the coupling of the spin state with the vector potential of the vortex beam. Since we consider a linearly polarized light in x-direction, the cross product is $[\hat{\sigma} \times \hat{\epsilon}]_z = -\sigma_y$. The photo-induced SOI transition matrix elements $\mathcal{S}_{n_j,n_i}^{\ell_j,\ell_i,s_j,s_i} = \langle s_j | \langle n_j \ell_j | \hat{H}_{\mathrm{SOI}} | n_i \ell_i \rangle | s_i \rangle = -\frac{\alpha}{\hbar} \langle n_j \ell_j | A_x(\boldsymbol{r},t) | n_i \ell_i \rangle \langle s_j | \sigma_y | s_i \rangle$ is given by

$$
\mathcal{S}_{n_j,n_i}^{\ell_j,\ell_i,s_j,s_i} = s_i \tilde{\alpha}\, h_{n_j,n_i}^{\ell_j,\ell_i} \delta_{\ell_j-\ell_i,m_{\mathrm{OAM}}} \delta_{s_j,-s_i}, \tag{9}
$$

where $\tilde{\alpha} = i A_0 e \alpha_R/\hbar$ and the radial integral is $h_{n,n'}^{\ell,\ell'} = \int_0^\infty \mathrm{d}r\, R_{n'\ell'}(r) R_{n\ell}(r) f_{m_{\mathrm{OAM}}}(r)$. This laser-induced interaction leads to spin-flip transitions within one spin channel as well as between the two different channels $(+)$ and $(-)$ due to $\langle s_j|\sigma_y|s_i\rangle$ (in the sub-states). Further, the (internal) orbital angular momentum state is changed due transfer of orbital angular momentum.

The electric scalar potential satisfies the Lorenz gauge conditions $\Phi(\boldsymbol{r},t) = -c^2 \int_{-\infty}^t \mathrm{d}t'\, \nabla \cdot \boldsymbol{A}(\boldsymbol{r},t')$ and gives rise to matrix elements of the form $\mathcal{K}_{n_j,n_i}^{\ell_j,\ell_i} = \langle n_j \ell_j | H_{\mathrm{el}} | n_i \ell_i \rangle$ (the spin orientation is again conserved):

$$
\begin{aligned}
\mathcal{K}_{n_j,n_i}^{\ell_j,\ell_i} = & \frac{2eA_0}{\omega_x c^2} \sum_\lambda \left[\frac{1}{w_0^3} u_{n_j,n_i}^{\ell_j,\ell_i+\lambda} - \frac{\lambda m_{\mathrm{OAM}}}{w_0} v_{n,n'}^{\ell,\ell'+\lambda} \right] \\
& \times \delta_{\ell_j-\ell_i,m_{\mathrm{OAM}}+\lambda}.
\end{aligned} \tag{10}
$$

This light-induced transition initiated by the electric scalar potential $\Phi(\boldsymbol{r},t)$ is comparable to the direct interaction $\hat{H}_D$ in Eq. (7). Thus, indirect spin-flip transitions are possible due to orbital couplings between both spin channels. Furthermore, the internal orbital angular momentum state changes during the interaction.

References

1. Weiss, U. *Quantum Dissipative Systems* (World Scientific, Singapore, 1999).

2. Chirolli, L. & Burkard, G. Decoherence in solid-state qubits. *Adv. Phys.* **57**, 225–285, DOI: 10.1080/00018730802218067 (2008).

3. Brandes, T. & Vorrath, T. Adiabatic transfer of electrons in coupled quantum dots. *Phys. Rev. B* **66**, 075341, DOI: 10.1103/PhysRevB.66.075341 (2002).

4. Thorwart, M. & Hänggi, P. Decoherence and dissipation during a quantum xor gate operation. *Phys. Rev. A* **65**, 012309, DOI: 10.1103/PhysRevA.65.012309 (2001).

5. Stavrou, V. & Hu, X. Charge decoherence in laterally coupled quantum dots due to electron-phonon interactions. *Phys. Rev. B* **72**, 075362, DOI: 10.1103/PhysRevB.72.075362 (2005).

6. Brandes, T. & Kramer, B. Spontaneous emission of phonons by coupled quantum dots. *Phys. Rev. Lett.* **83**, 3021, DOI: 10.1103/PhysRevLett.83.3021 (1999).

7. Murdin, B. *et al.* Time resolved studies of intersubband relaxation in gaas/algaas quantum wells below the optical phonon energy using a free electron laser. *Superlattices Microstruct.* **19**, 17–24, DOI: 10.1006/spmi.1996.0003 (1996).
